# Supplementary figures and images for: Quantitative analysis of the impact of climate variability and human activities on grassland productivity of the Qilian Mountain National Park, China
Source: PLoS One. 2024 May 10;19(5):e0300577. doi: 10.1371/journal.pone.0300577 (PMC11086858; doi:10.1371/journal.pone.0300577)

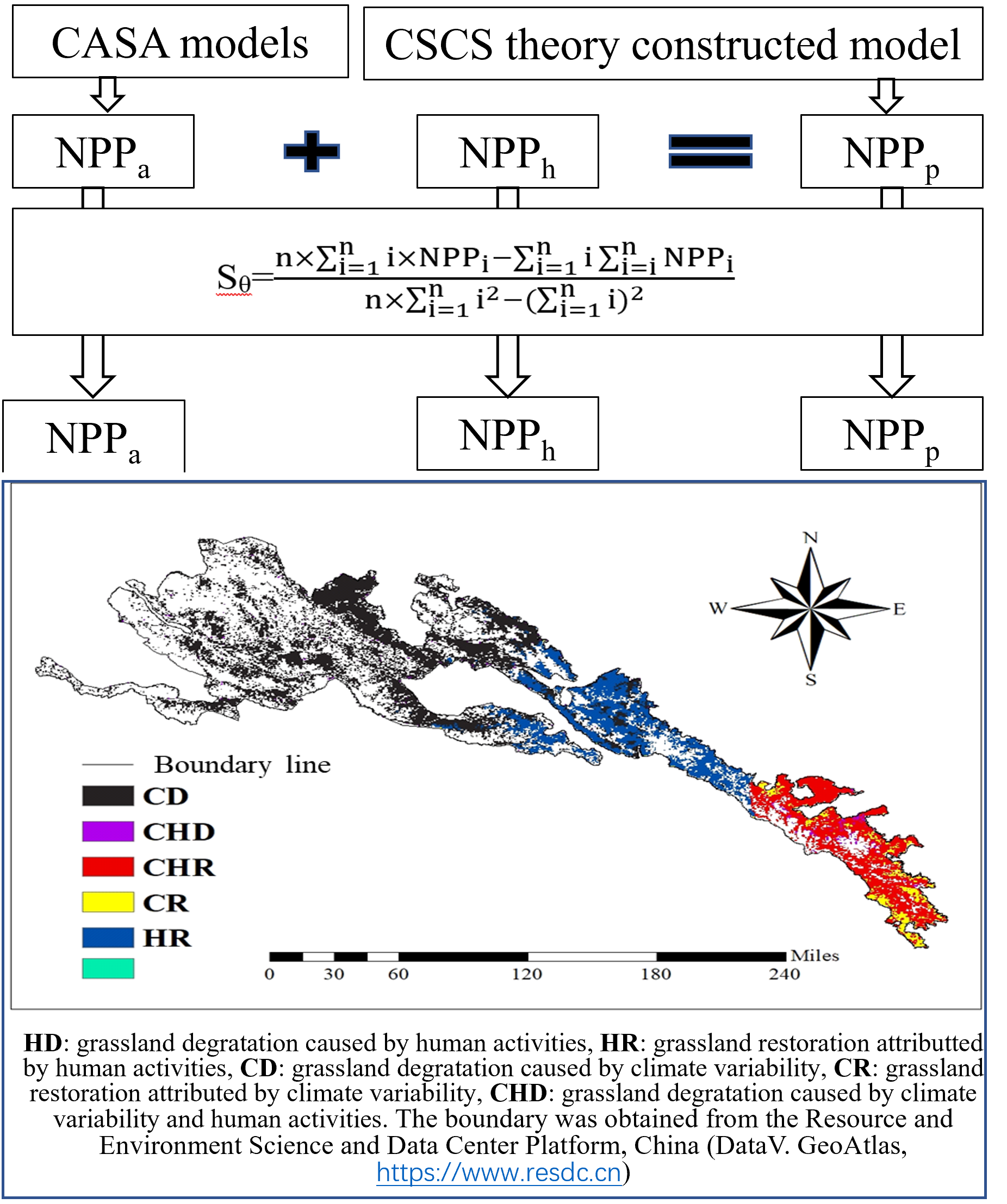

Supplement: S1 Fig — (PNG) [file pone.0300577.s001.png]
