# Supplementary material for: Quantitative analysis of the impact of climate variability and human activities on grassland productivity of the Qilian Mountain National Park, China
Source: PLoS One. 2024 May 10;19(5):e0300577. doi: 10.1371/journal.pone.0300577 (PMC11086858; doi:10.1371/journal.pone.0300577)
Supplement: Attachment — Submitted filename: Response to Reviewers.doc [file pone.0300577.s002.doc]

**RESPONSES TO EDITORS AND REVIEWERS**

**Aug 22, 2023**

**Tunira Bhadauria, Ph.D.**

**Academic Editor**

**PLOS ONE**

**Dear Dr.** **Tunira Bhadauria, Ph.D.:**

Thank you very much for your email and for the reviewers” **Quantitative analysis of the impact of climate variability and human activities on grassland productivity of the Qilian Mountain National Park, China (PONE-D-23-17536)**”. The comments were all valuable and very helpful in revising and improving our paper; these comments are also important guidance that is significant to our research. We studied the comments carefully and made corrections that we hope meet with your approval. We tried our best to improve the manuscript and made changes to the manuscript accordingly. These changes do not influence the content or framework of the paper. Once again, thank you very much for your comments and suggestions.

The main corrections in the paper and the responses to the reviewer comments are as follows:

**Response to Journal Requirements:**

1. Please ensure that your manuscript meets PLOS ONE's style requirements, including those for file naming. The PLOS ONE style templates can be found at

https://journals.plos.org/plosone/s/file?id=wjVg/PLOSOne_formatting_sample_main_body.pdf and

<https://journals.plos.org/plosone/s/file?id=ba62/PLOSOne_formatting_sample_title_authors_affiliations.pdf>

Response: we had amended.

2. Please note that funding information should not appear in any section or other areas of your manuscript. We will only publish funding information present in the Funding Statement section of the online submission form. Please remove any funding-related text from the manuscript.

Response: we had amended.

3. We note that the grant information you provided in the ‘Funding Information’ and ‘Financial Disclosure’ sections do not match.

When you resubmit, please ensure that you provide the correct grant numbers for the awards you received for your study in the ‘Funding Information’ section.

Response: we had amended.

4. Thank you for stating the following financial disclosure: "no"

Please state what role the funders took in the study. If the funders had no role, please state: "The funders had no role in study design, data collection and analysis, decision to publish, or preparation of the manuscript."

If this statement is not correct you must amend it as needed.

Please include this amended Role of Funder statement in your cover letter; we will change the online submission form on your behalf.

Response: we had amended

5. In your Data Availability statement, you have not specified where the minimal data set underlying the results described in your manuscript can be found. PLOS defines a study's minimal data set as the underlying data used to reach the conclusions drawn in the manuscript and any additional data required to replicate the reported study findings in their entirety. All PLOS journals require that the minimal data set be made fully available. For more information about our data policy, please see <http://journals.plos.org/plosone/s/data-availability>. "Upon re-submitting your revised manuscript, please upload your study’s minimal underlying data set as either Supporting Information files or to a stable, public repository and include the relevant URLs, DOIs, or accession numbers within your revised cover letter. For a list of acceptable repositories, please see [http://journals.plos.org/plosone/s/data-availability#](http://journals.plos.org/plosone/s/data-availability) loc-recommended-repositories. Any potentially identifying patient information must be fully anonymized.

Important: If there are ethical or legal restrictions to sharing your data publicly, please explain these restrictions in detail. Please see our guidelines for more information on what we consider unacceptable restrictions to publicly sharing data: [http://journals.plos.org/plosone/s/data-availability#loc-unacceptable](http://journals.plos.org/plosone/s/data-availability#loc-unacceptable) -data-access-restrictions. Note that it is not acceptable for the authors to be the sole named individuals responsible for ensuring data access.

We will update your Data Availability statement to reflect the information you provide in your cover letter.

Response: we had amended

6. We note that Figures 1,3 and 8 in your submission contain map/satellite images which may be copyrighted. All PLOS content is published under the Creative Commons Attribution License (CC BY 4.0), which means that the manuscript, images, and Supporting Information files will be freely available online, and any third party is permitted to access, download, copy, distribute, and use these materials in any way, even commercially, with proper attribution. For these reasons, we cannot publish previously copyrighted maps or satellite images created using proprietary data, such as Google software (Google Maps, Street View, and Earth). For more information, see our copyright guidelines: http://journals.plos.org/plosone/s/licenses-and-copyright.

We require you to either (a) present written permission from the copyright holder to publish these figures specifically under the CC BY 4.0 license, or (b) remove the figures from your submission:

a. You may seek permission from the original copyright holder of Figures 1,3 and 8 to publish the content specifically under the CC BY 4.0 license.

We recommend that you contact the original copyright holder with the Content Permission Form (http://journals.plos.org/plosone/s/file?id=7c09/content-permission-form.pdf) and the following text:

“I request permission for the open-access journal PLOS ONE to publish XXX under the Creative Commons Attribution License (CCAL) CC BY 4.0 (http://creativecommons.org/licenses/by/4.0/). Please be aware that this license allows unrestricted use and distribution, even commercially, by third parties. Please reply and provide explicit written permission to publish XXX under a CC BY license and complete the attached form.”

Please upload the completed Content Permission Form or other proof of granted permissions as an "Other" file with your submission.

In the figure caption of the copyrighted figure, please include the following text: “Reprinted from [ref] under a CC BY license, with permission from [name of publisher], original copyright [original copyright year].”

b. If you are unable to obtain permission from the original copyright holder to publish these figures under the CC BY 4.0 license or if the copyright holder’s requirements are incompatible with the CC BY 4.0 license, please either i) remove the figure or ii) supply a replacement figure that complies with the CC BY 4.0 license. Please check copyright information on all replacement figures and update the figure caption with source information. If applicable, please specify in the figure caption text when a figure is similar but not identical to the original image and is therefore for illustrative purposes only.

The following resources for replacing copyrighted map figures may be helpful:

USGS National Map Viewer (public domain): http://viewer.nationalmap.gov/viewer/

The Gateway to Astronaut Photography of Earth (public domain): http://eol.jsc.nasa.gov/sseop/clickmap/

Maps at the CIA (public domain): https://www.cia.gov/library/publications/the-world-factbook/index.html and https://www.cia.gov/library/publications/cia-maps-publications/index.html

NASA Earth Observatory (public domain): http://earthobservatory.nasa.gov/

Landsat: http://landsat.visibleearth.nasa.gov/

USGS EROS (Earth Resources Observatory and Science (EROS) Center) (public domain): http://eros.usgs.gov/#

Natural Earth (public domain): http://www.naturalearthdata.com/

[Note: HTML markup is below. Please do not edit.]

Response: we had amended. Such as, “The maps of Qilian Mountain National Park and China (inspection number: GS Jing (2022) 1061) were downloaded from the Resource and Environment Science and Data Center Platform, China (DataV. GeoAtlas, https://www.resdc.cn/data.aspx?DATAID=272) and using ArcGIS 10.2.2 platform split (ESRI, Redlands, CA: Environmental Systems Research Institute)”.

**Response to Editor comments (Tunira Bhadauria, Ph.D):**

Please use the space provided to explain your answers to the questions above. You may also include additional comments for the author, including concerns about dual publication, research ethics, or publication ethics. (Please upload your review as an attachment if it exceeds 20,000 characters)

Response: we had provided to explain your answers to the questions above.

**Response to Reviewer #1**

Manuscript entitled "Quantitative analysis of the impact of climate variability and human activities on

grassland productivity of the Qilian Mountain National Park, China" are recommended for publication. Although following modification are required

1.Future perspective must be included in conclusion

Response: we had amended.

2.An abstract figure which may summaries whole work may be drawn

Response: we had amended.

**Response to Reviewer #2:**

Dear Author, The manuscript Quantitative analysis of the impact of climate variability and human activities on

grassland productivity of the Qilian Mountain National Park, China has been written well and looks sound. However, some modifications are needed before publishing the MS.

Introduction part is bit large so i would suggest to write it in more crisp way to attract the readers.

Response: we had amended

In results section, figure 3 4 and figure 6 A, it is difficult to understand. For example figure 3, the Spatial distribution of the monthly grassland NPP has been shown there from January to December but the year 2000 to 2015 is not reflecting in the figure. Kindly look into this and explain if it is okay or make corrections if needed.

Response: we had amended.

The number of figures are too much in the MS, therefore, shift some figures in the supplementary figures. Check the fig. 6 where it is mentioned that 2000 and 2015, it must be 2000 to 2015.

Response: we had amended.

The discussion is still not that much supportive to your findings.

Response: we had amended.

Better to rewrite it giving some more conclusive remarks by supporting your findings. At this stage its like review of literature.

Response: we had amended.

After these small corrections, the manuscript is recommended for publication.

In all we feel that these results are interesting and that all the parts to a focused manuscript are in the authors possession, they just need to refocus the paper and make sure that the purpose of the study, and discussion points are all clearly linked together and the results are put into a broader context.

We tried our best to improve the manuscript and made changes to the manuscript accordingly. These changes do not influence the content or framework of the paper. We appreciate the Editors’ and Reviewers’ earnest help, and hope that the corrections are sufficient. Once again, thank you very much for your comments and suggestions.

Yours sincerely,

Qiang Li

Corresponding author

E-mail: 1245524440@qq.com
